# A novel Pt(iv) mono azido mono triazolato complex evolves azidyl radicals following irradiation with visible light†
†Electronic supplementary information (ESI) available: Synthetic details and characterisation data including X-ray crystallographic tables. CCDC 1883808. For ESI and crystallographic data in CIF or other electronic format see DOI: 10.1039/c9dt01156k


**DOI:** 10.1039/c9dt01156k

**Published:** 2019-04-23

**Authors:** Kezi Yao, Arnau Bertran, Jacques Morgan, Samuel M. Hare, Nicholas H. Rees, Alan M. Kenwright, Katharina Edkins, Alice M. Bowen, Nicola J. Farrer

**Affiliations:** a Chemistry Research Laboratory , University of Oxford , 12 Mansfield Road , Oxford , OX1 3TA , UK . Email: Nicola.Farrer@chem.ox.ac.uk ; Tel: +44 (0)1865 285131; b School of Pharmacy , Queen's University Belfast , 97 Lisburn Road , Belfast BT9 7BL , UK; c Department of Chemistry , University of Durham , South Road , Durham DH1 3LE , UK

## Abstract

The platinum(iv) azido complex *trans*,*trans*,*trans*-[Pt^IV^(N_3_)_2_(OH)_2_(py)_2_] (**1**) undergoes cycloaddition with 1,4-diphenyl-2-butyne-1,4-dione (**2**) under mild, catalyst-free conditions, affording a number of mono and bis click products. The major mono click product (**3**) exists in MeCN as an equilibrium mixture between two species; **3a** and **3b** rapidly interconvert through nucleophilic attack of the axial Pt–OH group at the adjacent Ph–CO group. The kinetic and thermodynamic parameters for this interconversion have been measured by selective saturation-transfer NMR spectroscopic experiments and are consistent with cyclisation at the Pt centre. Complex **3b** was also characterised by X-ray crystallography. Visible light irradiation (440–480 nm) of **3** in *d*_3_-MeCN produces azidyl radicals (N_3_˙), as demonstrated by EPR spin-trapping with DMPO; no generation of hydroxyl radicals was observed. ^1^H–^195^Pt HMBC NMR confirmed that the photoproducts were Pt^IV^ rather than Pt^II^ species, and HPLC was consistent with these being [**3**–N_3_]^+^ species; no facile photoejection of the triazolato ligand was observed, consistent with MS/MS fragmentation of **3**. When **3** was irradiated in the presence of 5′-GMP, no 5′-GMP photoproducts were observed, suggesting that complex **3** is likely to exhibit significantly simplified biological activity (release of azidyl radicals but not DNA binding) compared with complex **1**.

## Abstract 2

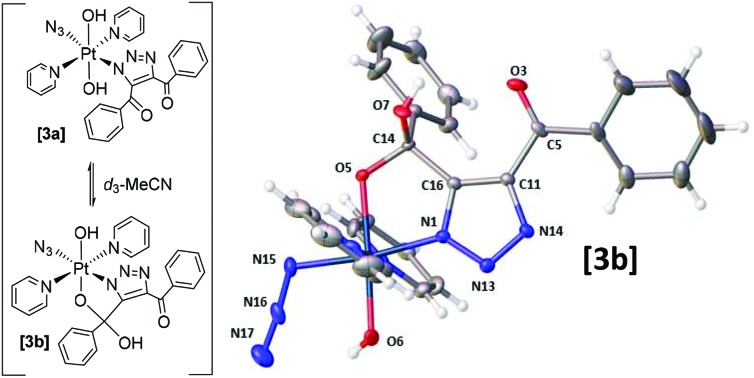
A novel Pt^IV^ azido triazolato complex exists as an equilibrium between two species in *d*_3_-MeCN and evolves azide radicals (but not hydroxide radicals) when irradiated with visible light.

## Introduction

Reactivity of metal azides towards acetylenes in a [3 + 2] 1,3-dipolar cycloaddition (or “click” reaction) to form triazoles is a well-established route to form new metal complexes and new ligands which are not readily accessible by other routes.^1^ Although [3 + 2] cycloadditions have been extensively investigated in organic synthesis, the potential of cycloaddition reactions of metal-coordinated ligands is less well-explored. To date, click reactions between metal azides and alkynes (or masked alkynes)^2^ have been used in a range of synthetic investigations; forming Pt(ii)–Au(i) heterometallic arrays,^3^ tri-Au(i) triazoles,^4^ Ru-based catalysts^5^ and metalloenzyme inhibitors.^6^ They have also been used to synthesise Au(i) triazole peptide conjugates targeted to mitochondria, which showed promising anti-cancer activity.^7^ The new triazole ligands formed in these cycloaddition reactions themselves have a wide-range of chemistries and potential applications.^8^ Whilst copper(i)-catalysed (CuAAC) reactions dominate the literature,^9^ our focus is on copper-free cycloadditions, since the cytotoxicity of copper salts is undesirable for biological applications.^10^ We recently reported that *trans*,*trans*,*trans*-[Pt(N_3_)_2_(OH)_2_(py)_2_] **1** is reactive towards a number of strained cyclooctynes and highly electron-deficient acetylenes including DMAD, but does not react with other acetylenes including phenylpropiolic acid, 1-phenyl-2-propyn-1-ol and 3-butyn-2-one.^11^

We focused on internal alkynes, because terminal alkynes (HC≡CR) often undergo azide-acetylene ligand exchange with metal azides, rather than 1,3-dipolar cycloaddition; for example, *cis*-[Pt(N_3_)_2_(PPh_3_)_2_] reacts with acetylenylbenzene to give *trans*-[Pt(C≡CPh)_2_(PPh_3_)_2_].^12^ Consequently, we investigated the reactivity of the azido complex **1** towards the acetylene 1,4-diphenyl-2-butyne-1,4-dione (**2**) and here we report our findings.

**Scheme 1:**
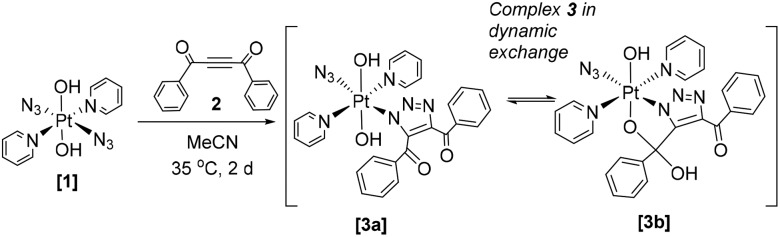
Reaction of *trans*,*trans*,*trans*-[Pt(N_3_)_2_(OH)_2_(py)_2_] (**1**) with 1,4-diphenyl-2-butyne-1,4-dione (**2**) showing formation of the major product **3** (**3a**/**3b**).

Although no Pt(ii) or Pt(iv) examples have been previously reported, acetylene **2** (Scheme 1) undergoes cycloadditions with both Pd(ii)^13^ and Ni(ii)^14^ mono azido complexes. For Pd(ii), the coordinated triazole ligand is not constrained through additional cyclisation, and equivalent ^1^H NMR spectral resonances for the Ph groups were observed, consistent with rearrangement to N2 triazole coordination. For Ni(ii), in addition to triazole formation, one of the PhCO groups of the triazole condensed with a ligand-based NH_2_ group, forming a 5-membered ring containing the Ni; the IR and ^1^H NMR spectra indicated triazole formation and retention of N1-coordination. It is generally accepted that the N1-bound triazole isomer forms as the kinetic product of the cycloaddition reaction. For metal-based cycloadditions – in contrast to purely organic cycloadditions – this isomer may often then undergo rearrangement to the more thermodynamically stable N2-bound isomer.^15^ The N1/N2 coordination preference appears to be a balance of sterics, electronics and any potential ligand cyclisation which could “lock” the triazole in N1 coordination. Small modifications in the ligands may alter the N1/N2 preference; for the reaction of Ru-azido complexes with DMAD and diethylacetylenedicarboxylate (DEACD), X-ray crystallography confirmed a change in preference from N1 (ethyl) to N2 (methyl) triazole coordination depending on the ligand substituents for a number of Ru azido complexes.^16^,^17^

Since Pt(iv) azido groups are considerably less electron-rich than Pt(ii) azides they are anticipated to be even less amenable to cycloaddition with electron-deficient acetylenes. We were therefore surprised to observe the reaction of **2** with the Pt^IV^ diazido complex **1** under mild conditions.

## Results and discussion

After stirring a solution of **1** and **2** (1.1 eq.) in MeCN (35 °C, 2 d) the reaction solution was concentrated under vacuum and filtered. RP-HPLC (MeCN + 0.1% NH_4_OH/H_2_O + 0.1% NH_4_OH, pH 9) purification of the filtrate revealed four new cycloaddition products, three mono-substituted species; (*t*_R_ = 4.01 min, 8%); complex **3** (*t*_R_ = 4.31 min, 73%); (*t*_R_ = 5.44 min, 2%) and one bis-substituted species (*t*_R_ = 5.94 min, 7%) (Fig. S1†).

### Complex **3** consists of interconverting isomers **3a** and **3b**

**Fig. 1:**
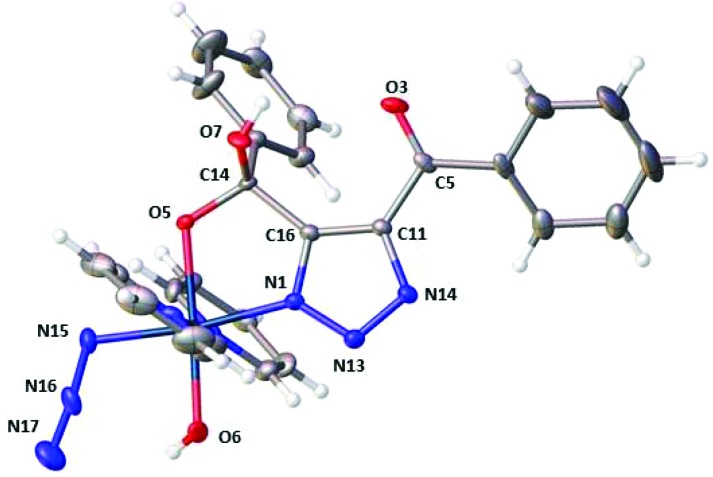
X-Ray crystallographic structure of **3b** with thermal ellipsoids displayed at 50% probability.^18^ Pt-Azido group naming convention; N15 = N_α_, N16 = N_β_ and N17 = N_γ_.

HPLC solvent was removed from the main product **3** (*t*_R_ 4.3 min, 73%) by freeze-drying; yellow crystals were grown by vapour diffusion of tetrahydropyran into a concentrated MeCN solution of **3**. Single crystal X-ray crystallography revealed the structure of the mono-click product (**3b**) which had cyclised *via* attack of the Pt–OH ligand at the PhCO group to form a 5-membered ring (Fig. 1).

Both the azido (Pt–N_α_–**N**_**β**_–N_γ_) ligand angle and bond lengths are very similar to those seen in the diazido precursor complex; (174.8(6)° in **3b***vs.* 174.4(4)/175.3(4)° in **1**);^19^ and 1.218(6)/1.215(4), 1.218(5), Å (N_α_–N_β_) and 1.142(6)/1.139(4), 1.146(5) Å (N_β_–N_γ_) in **3b** and **1** respectively. The angle subtended at Pt–**N**_**α**_–N_β_ is more acute for complex **3b** (114.2(3)°) compared to complex **1** (118.0(3)°, 120.3(2)°). The Pt–N1 triazole bond length is 1.998(4) Å, which compared to reported Pt^II^ N1-coordinated 1,4-disubstituted triazole complexes with lengths of 2.149(2) Å–2.139(2) Å, is indicative of relatively strong bonding.^8^,^20^

Although on re-injection for analytical HPLC, **3** eluted as a single peak (*t*_R_ = 4.40 min) (Fig. S2†), in *d*_3_-MeCN solution at 25 °C, **3** existed in dynamic equilibrium between two isomers – **3a** and **3b** – in a 1 : 1 ratio. ^1^H and ^13^C NMR spectral resonances of complexes **3a** and **3b** were fully characterised by 1D and 2D (COSY/ROESY/HSQC/HMBC) spectroscopic techniques (Fig. S3†).

**Fig. 2:**
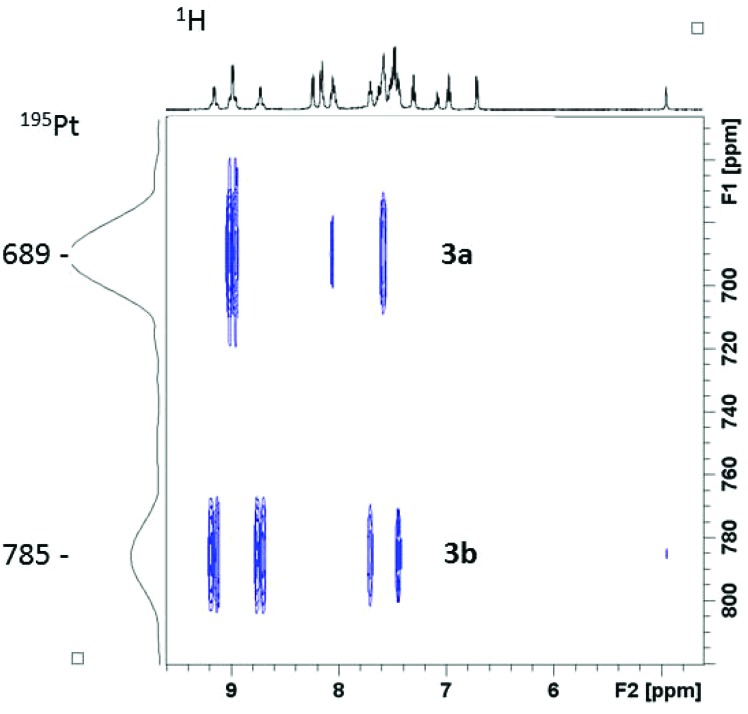
^1^H–^195^Pt HMBC NMR (proton at 500 MHz) spectrum of complex **3** in *d*_3_-MeCN, showing the presence of two isomers **3a** and **3b**.

^1^H–^195^Pt HMBC NMR spectroscopy revealed two distinct ^195^Pt environments, for **3a** (689 ppm) and **3b** (785 ppm) respectively (Fig. 2). Addition of D_2_O to the *d*_3_-MeCN NMR sample resulted in the loss of the **3b** C-OH ^1^H NMR spectral resonance at 4.93 ppm, as a result of proton/deuterium exchange. The equilibrium species **3a**/**3b** were stable in *d*_3_-MeCN (in the dark), for a period of at least 6 months.

Depending on the mass spectrometer, either Na^+^ or H^+^ adducts of **3** were also observed by ESI-MS; [(**3**)_2_ + Na]^+^ was detected at 1433.29 *m*/*z* and [**3** + Na]^+^ at 728.14 *m*/*z*; and under some conditions [**3** + H]^+^ was detected at 706.15 *m*/*z*. The [**3**–OH]^+^ adduct at 688.15 *m*/*z* was consistently observed on all instruments (Fig. S4†). HRMS of **3** supported the identity of both [**3** + H]^+^ (706.1438 *m*/*z*) (Fig. S5†) and [**3**–OH]^+^ (688.1379 *m*/*z*) (Fig. S6†) species. MS/MS fragmentation of the [**3** + H]^+^ species (706.15 *m*/*z*) revealed that it very readily fragmented to give [**3**–OH]^+^ (688.14 *m*/*z*, C_26_H_21_N_8_O_3_Pt calc. 688.14 *m*/*z*) and also the [Pt(triazole)(py)(OH)]^+^ species (567.09 *m*/*z*, C_21_H_16_N_4_O_3_Pt calc. 567.09 *m*/*z*) through loss of pyridine and azide (where triazole = C_16_H_10_N_3_O_2_) (Fig. S4†).

The [**3**–triazole]^+^ fragment ([Pt(OH)_2_Py_2_(N_3_)]^+^*ca.* 429.06 *m*/*z*) was not observed, indicating that the triazole ligand does not preferentially dissociate to give a stable charged species without other ligands also being lost; however, smaller fragments such as [Pt(OH)(py)_2_]^+^ (370.0546 *m*/*z*, C_10_H_11_N_2_OPt, calc. 370.0519 *m*/*z*) and [Pt(N_3_)(triazole)]^+^ (513.0912 *m*/*z*, C_16_H_10_N_6_O_2_Pt, calc. 513.0513 *m*/*z*) were detected. MS/MS of the [**3**–OH]^+^ 688.14 *m*/*z* ion gave identical daughter ions and fragmentation behaviour to MS/MS of the [**3** + H]^+^ (706.15 *m*/*z*) species.

UV-Vis spectroscopy of **3** was consistent with partial loss of a N_3_ → Pt LMCT band, with *λ*_max_ moving to shorter wavelengths, from 299.5 nm (**1**) to 261.5 nm (**3**) (Fig. S7†). IR spectroscopy of a freshly prepared *d*_4_-MeOH sample of **3** (Fig. S8†) showed the strong *ν*_asym_N_3_ stretch at 2048 cm^–1^ was slightly weaker compared to the starting diazido complex **1** (2051 cm^–1^, solid)^21^ but similar to the value we reported for the cyclometallated mono triazole DMAD complex *trans*,*trans*,*trans*-[Pt(N_3_)(C_5_H_3_N_3_O_4_)(OH)(py)_2_] at 2046 cm^–1^ (solid).^11^

### Interconversion kinetics of **3a**/**3b**

The kinetics of the **3a**/**3b** equilibrium were investigated *via* selective saturation-transfer experiments on interconverting ^1^H NMR spectroscopic signals at 6.94 ppm (**3b**_Phmeta_) and 7.28 ppm (**3a**_Phmeta_) in *d*_3_-MeCN.^22^ Data was acquired at 9 temperature points; the final values for the thermodynamic parameters were calculated using an Eyring plot (Fig. S9†): Δ*G*^‡^ = 76 (1) kJ mol^–1^ (at 298 K); Δ*S*^‡^ = –173 (4) J mol^–1^ K^–1^, and Δ*H*^‡^ = 25 (1) kJ mol^–1^ (error in brackets). The exchange rate constant *k* was determined as 0.28 s^–1^ (298 K), consistent with similar processes.^23^ The negative entropic change is consistent with cyclisation of **3a** to **3b**. We were unable to find reports of any directly comparable systems involving cyclisation of a ligand which is attached to a Pt centre, rather than bond breaking/formation at the metal centre itself. However, the activation barrier appears to be in the appropriate range for a reversible transformation at a metal centre on the NMR timescale, and values reported for reversible bond formation and breakage at a Pt^IV^ centre^24^ and rotation of a Pt^II^–allene complex^25^ are consistent with our findings.

### Photochemistry of **3**

**Fig. 3:**
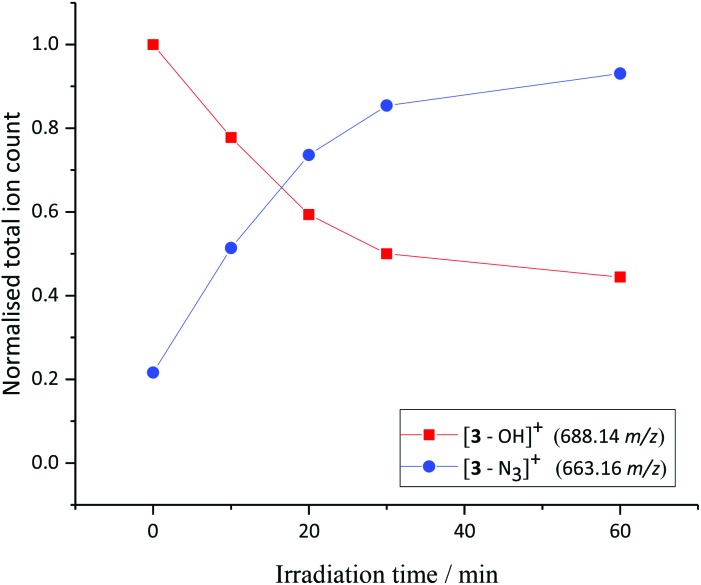
Irradiation of **3** (detected by ESI-MS predominantly as [**3**–OH]^+^) in the presence of 5′-GMP with UVA irradiation in PBS solution; resulted in photorelease of N_3_ and formation of [**3**–N_3_]^+^, as monitored by HPLC.

A solution of **3** and 5′-GMP (5 equiv.) in phosphate-buffered saline (PBS) solution was irradiated with UVA light and the reaction monitored by LCMS (Fig. 3). Photochemically-induced azide release was observed, through detection of the [**3**–N_3_]^+^ fragment (663.16 *m*/*z*) in the mass spectrometer. The identity of this main photoproduct was confirmed by HRMS (663.1318 *m*/*z* measured, C_26_H_22_N_5_O_4_Pt, calc. 663.1325 *m*/*z*, Fig. S11†).

The photochemistry of **3** was also investigated by ^195^Pt NMR spectroscopy in *d*_3_-MeCN. Following irradiation (452 nm, 1 h), new Pt^IV^ signals were observed, but no new ^195^Pt signals were observed in the Pt^II^ region of the spectrum. This supports the hypothesis that upon irradiation, **3** does not form Pt^II^ photoproducts, unlike for the precursor diazido complex **1** which forms a variety of photoproducts, including Pt^II^ species. We previously reported a range of adducts formed when **1** is irradiated in the presence of 5′-GMP.^19^ In contrast, when complex **3** was irradiated in the presence of 5′-GMP, no adducts were detected by ESI-MS. Pt^IV^ diazido complexes have demonstrated evolution of azidyl and hydroxyl radicals under irradiation, which is thought to contribute to their potent photocytotoxic effects.^26^–^28^ EPR experiments in nitrogen-saturated MeCN solutions confirmed production of azidyl radicals under blue light irradiation (440–480 nm) by trapping with DMPO to form the DMPO–N_3_˙ adduct, which quickly built up a steady-state concentration of 18 μM, and then decayed on further illumination. This is consistent with our previous results from irradiation of Pt^IV^ azido complexes.^27^ Although no hydroxyl radicals were detected through trapping with DMPO to form DMPO–OH˙, small quantities (4 μM) of a stable non-coupled nitroxide species was formed on a timescale significantly slower than the formation of DMPO–N_3_˙. It was not detected when DMPO alone was irradiated in nitrogen-saturated MeCN. It is known that azide spin-adducts can themselves be transformed *via* S_N_1 chemistry into other secondary species;^29^ further investigation is ongoing (Fig. S12–S14†).

## Experimental

For materials, methods and procedures see ESI.†

## Conclusions

The Pt^IV^ mono azido mono triazolato complex product (complex **3**) of the reaction between **1** and **2**, has been fully characterised. To the best of our knowledge, the isomer **3b** is the first reported X-ray crystal structure of a Pt(iv) mono azido complex. In MeCN, complex **3** exists as an equilibrium mixture of open **3a** and cyclised **3b** isomers in 50 : 50 ratio; the thermodynamics of this interconversion are consistent with ring-opening and closing. Complex **3** can be photoactivated with UVA (365 nm) and visible (452 nm) light in solution to give [Pt^IV^–N_3_]^+^ as the main photoproduct, as determined by ESI-MS. EPR experiments of an MeCN solution of **3** under irradiation (440–480 nm) confirmed production of azidyl radicals through spin-trapping with DMPO, as seen previously for the precursor diazido species **1**. No OH radicals were trapped when **3** was irradiated, consistent with a [**3**–N_3_]^+^ species being the major photoproduct.

We did not detect photoejection of the triazole ligand from the Pt centre of **3** in any of our irradiation experiments, possibly due to the additional stability afforded by cyclisation of the biphenyl ligand, resulting in bidentate coordination. MS/MS studies of **3** also confirmed the strong binding of the triazole ligand. Irradiation of **3** in the presence of the DNA model 5′-GMP with either UVA or 452 nm light did not produce any detectable 5′-GMP-Pt products.

The photocytotoxic mechanisms of diazido complexes such as **1** are complicated, since azidyl radicals, hydroxyl radicals, ^1^O_2_, various DNA-binding Pt^IV^ and Pt^II^ fragments can be produced upon irradiation.^28^ The relatively simple photochemistry observed for complex **3** may enable us to better understand the photocytotoxic role and relative importance of the azidyl radical when released *in cellulo* – these photobiological experiments are ongoing. Such azidyl-releasing complexes in the same line as photoCORMS (CO-releasing)^30^ and NORMS (NO releasing)^31^ complexes may find application as phototherapeutics since azidyl radicals and azide anions are cytotoxic; one pathway by which azidyl radicals may exert their cytotoxic activity is *via* oxidative attack of tryptophan residues in proteins;^28^ and sodium azide inhibits cytochrome oxidase in the mitochondrial electron transport chain causing cell death.^32^

## Conflicts of interest

There are no conflicts to declare.

## Supplementary Material

Supplementary information

Crystal structure data
