# Supplementary material for: A novel Pt(iv) mono azido mono triazolato complex evolves azidyl radicals following irradiation with visible light
Source: Dalton Trans. 2019 Apr 23;48(19):6416–20. doi: 10.1039/c9dt01156k (PMC6984332; doi:10.1039/c9dt01156k)
Supplement: Supplementary file 1 [file DT-048-C9DT01156K-s001.pdf]

Electronic Supplementary Material (ESI) for Dalton Transactions.
This journal is © The Royal Society of Chemistry 2019

A novel Pt(IV) *mono* azido *mono* triazolato complex evolves azidyl radicals following irradiation with visible light

Electronic Supplementary Information

Kezi Yao, Arnau Bertran, Jacques Morgan, Samuel M. Hare, Nicholas H. Rees, Alan M. Kenwright, Katharina Edkins, Alice M. Bowen, Nicola J. Farrer

Contents

General procedures....1
Synthetic procedures....2
Characterisation of complex 3....4
ESI-MS, MS/MS and HRMS of complex 3 (3a/3b)....6
UV-Vis spectra....10
IR spectra....11
NMR kinetics....12
X-Ray crystallographic data and tables....14
Photochemistry of 3....17
EPR experimental data....18
References....22

General procedures

Materials and Methods. $K_2[PtCl_4]$ was purchased from Precious Metals Online. HPLC-grade solvents and Millipore-filtered H_2O were used for the preparation of compounds and purification by HPLC. DMPO (≥ 98 %) was purchased from Cambridge Bioscience Ltd. All other reagents were purchased from Sigma-Aldrich or Alfa Aesar and used as received. (IM) indicates use of a nylon syringe filter (pore size 0.2 μM). All manipulations were carried out under reduced lighting and solutions were prepared stored and handled with minimal exposure to light. **NMR spectroscopy.** Due to the potential photosensitivity of the compounds, amberised NMR spectroscopy tubes (Goss Scientific/Norrell) were used. Spectra were acquired at 298 K unless otherwise stated, and processed using Topspin 3.2. All chemical shift (δ) values are given in parts per million and are referenced to residual solvent unless otherwise stated, *J* values are quoted in Hz.**^{1}H and ^{195}Pt NMR:** were acquired on a Bruker AVIIIHD 500 MHz (500.13 MHz) equipped with a 5mm z-gradient broadband X-19F/1H BBFO SMART probe or a Bruker AVIIIHD 400 nanobay (400.17 MHz). **^{13}C NMR:** acquired on a Bruker AVII 500 MHz spectrometer equipped with a z-gradient triple resonance inverse ^{1}H/^{19}F(^{13}C) TXI probe. **^{1}H-^{195}Pt HMBC NMR spectra**: ^{195}Pt chemical shifts were externally referenced to

K_2PtCl_6 in 1.5 mM HCl in D_2O (δ 0 ppm). **Mass Spectrometry:** low resolution ESI-MS were obtained with a Waters Micromass LCT Premier XE spectrometer. **HRMS:** obtained with a Thermofisher Exactive Plus with a Waters Acuity UPLC system. **MS/MS experiments**: were performed on an Acuity UPLC in flow injection analysis mode, equipped with a Waters Xevo G25 QTOF. All MS data were processed using MassLynx 4.0. **HPLC:** were performed with a Waters Autopurification system, equipped with a Waters X-Bridge OBD semi-prep column (5 µm, 19 mm x 50 mm), with an injection loop of 1 ml, eluting with H_2O+0.1% NH_4OH (pH 9)/MeCN +0.1% NH_4OH. The crude samples (in H_2O/MeCN) were filtered (nylon, 0.2 µm) and injected in 750 µL aliquots, with mass-directed purification with an ACQUITY QDa performance mass spectrometer. Analytical HPLC used the same solvents and system, with a Waters X-Bridge OBD column (5µm, 4.6mm x 50mm) and an injection loop of 0.02 ml. Retention times (t_R) are quoted for the solvent gradient: 0 min (95% A : 5% B); 1 min (95:5), 7.5 min (5:95) on the analytical column. **UV-visible absorption spectra** were acquired with the Waters HPLC (Figure S7) or a UV-Vis spectrometer (Cary 60 UV-Vis, Agilent Technologies, Figure S12). **Photochemistry**: samples were irradiated with stirring at a distance of 50 mm from a MiniSun GU10 27 SMD LED bulb with an output centred at 452 nm (see **Figure S11**) or with a MiniSun UVA bulb.

Synthetic procedures

***Caution!* No problems were encountered during this work, however heavy metal azides are known to be shock sensitive detonators, therefore it is essential that platinum azides compound are handled with care.**

Trans, trans, trans-[$Pt^{IV}(N_3)_2(OH)_2(py)_2$] (**1**) was prepared from K_2PtCl_4 as previously reported and was purified by HPLC before use.[2]

Reaction of 1 with 1,4-diphenyl-2-butyne-1,4-dione (2) to give complex 3 (3a/3b).

1,4-diphenyl-2-butyne-1,4-dione (70 mg, 0.299 mmol) was dissolved in MeCN (22 ml). *Trans,trans,trans*-[$Pt(N_3)_2(OH)_2(py)_2$] (150 mg, 0.318 mmol) was added whilst stirring at 35 °C for 48 h. The solvent volume was reduced to 5 ml *in vacuo,* syringe filtered and purified by HPLC. HPLC revealed three *mono* substituted products at t_R = 4.0 min (**A, 5**), 4.3 min (major product, complex **3a/b**) and 5.4 min (**C**), and one *bis* substituted product t_R = 5.9 min (**bis**). Complexes **5**, **3a/b** and **C** were isolated and the solvent removed by freeze-drying. Complex **3a/3b** was isolated as a pale yellow solid (60 mg, 0.085 mmol, 28 %). Subsequent analysis of **3** confirmed that it was an equilibrium mixture of two isomers: **3a** and **3b**.

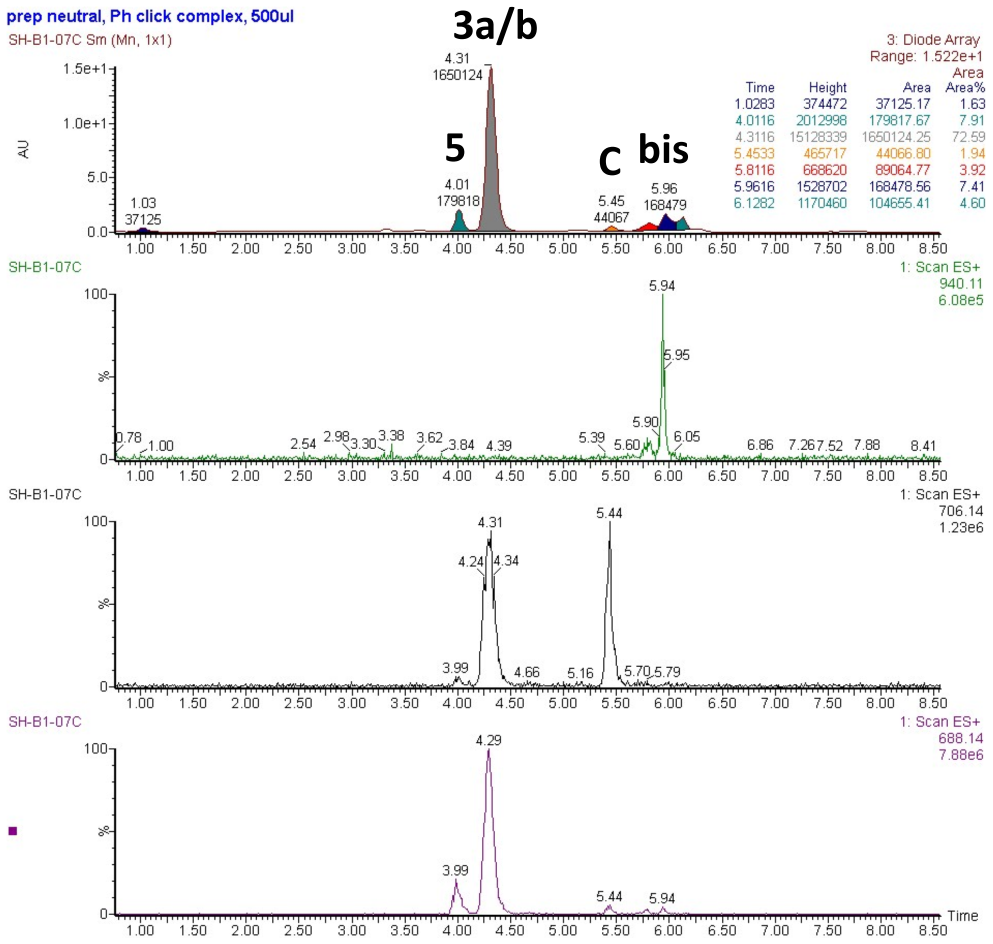

Figure S1. HPLC trace of crude mixture from reaction between complex **1** and **2** showing product distribution. t_R = 4.01 min (**5, 8%**), 4.31 min (complex **3a/3b, 73%**) and 5.44 min (**C, 2%**) and *bis* substituted product t_R = 5.94 min (**bis, 7%**). Unreacted starting material (**1**) is detected at 1.03 min. Relative product distributions are calculated by relative UV-Vis spectral absorbance integrated over the range 210 nm – 400 nm.

Characterisation of complex 3

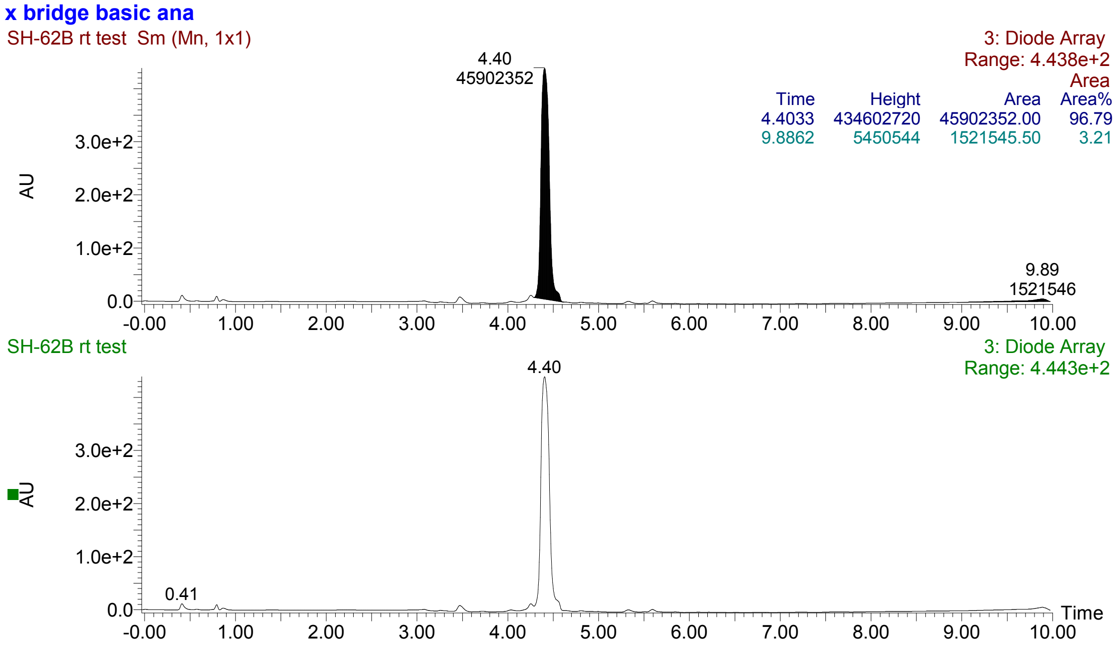

Figure S2. Analytical RP-HPLC trace of **3** showing co-elution of the two interconverting isomers **3a** and **3b**.

Complex 3 (3a/3b) (major product t_R= 4.3 min):

^{1}H NMR (500 MHz, d_3-MeCN) δ: 9.13 (dd, $^{3}J_{HPt}$ = 27.6, $^{3}J_{HH}$ = 6.7, 2H, **3b**$_{Pyortho}$), 8.95 (dd, $^{3}J_{HPt}$ = 28.0, $^{3}J_{HH}$ = 6.7, 4H, **3a**$_{Pyortho}$), 8.70 (dd, $^{3}J_{HPt}$ = 27.9, $^{3}J_{HH}$ = 6.7, 2H, **3b**$_{Pyortho'}$), 8.21 (d, $^{3}J_{HH}$ = 8.1, 2H, **3b**$_{Phortho'}$), 8.13 (d, $^{3}J_{HH}$ = 7.5, 2H, **3a**$_{Phortho'}$), 8.12 (m, 1H, **3b**$_{Pypara}$), 8.03 (m, 2H, **3a**$_{Pypara}$), 8.01 (m, 1H, **3b**$_{Pypara'}$), 7.68 (dd, 2H, **3b**$_{Pymeta}$), 7.60 (m, 1H, **3b**$_{Phpara'}$), 7.58, (m, 1H, **3a**$_{Phpara'}$), 7.56 (m, 4H, **3a**$_{Pyortho}$) 8.03, **(3a**$_{Pymeta}$), 7.49 (m, 1H, **3a**$_{Phpara}$), 7.47 (m, 2H, **3b**$_{Phmeta'}$), 7.46 (m, 2H, **3a**$_{Phmeta'}$), 7.45 (d, 2H, **3a**$_{Phortho}$), 7.43 (m, 2H, **3b**$_{Pymeta'}$), 7.26 (dd, $^{3}J_{HH}$ = 6.7, $^{3}J_{HH}$ = 6.7, 2H, **3a**$_{Phmeta}$), 7.06 (t, $^{3}J_{HH}$ = 7.5, 1H, **3b**$_{Phpara}$), 6.94 (dd, $^{3}J_{HH}$ = 7.6, $^{3}J_{HH}$ = 7.6, 2H, **3b**$_{Phmeta}$), 6.68 (d, $^{3}J_{HH}$ = 8.1, 2H, **3b**$_{Phortho}$), 4.93 (s, **3b**$_{C\text{-}OH}$).

^{13}C NMR (151 MHz, d_3-MeCN) δ: 190.3 (q, CO, **3a**$_{PhCO}$), 188.1 (q, CO, **3a**$_{PhCO'}$), 186.4 (q, CO, **3b**$_{PhCO}$'), 159.3 (q, $^{2}J_{CPt}$ = 48.6, **3b**$_{triazoleC4}$), 150.5 (**3b**$_{Pyortho'}$), 150.4 (**3b**$_{Pyortho}$), 150.1 (**3a**$_{Pyortho}$), 147.9 (q, $^{3}J_{CPt}$ = 25.8, **3b**$_{triazoleC5}$), 147.1 (q, $^{4}J_{CPt}$ = 13.6, **3b**$_{Phipso}$), 145.6 (q, $^{3}J_{CPt}$ = 33.1, **3a**$_{triazole4}$), 143.2 (**3b**$_{Pypara}$), 143.0 (**3b**$_{Pypara'}$), 142.9 ($^{4}J_{CPt}$ = 6, **3a**$_{Pypara}$), 142.2 (q, $^{2}J_{CPt}$ = 33.7, **3a**$_{triazole5}$), 138.1 (q, **3a**$_{Phipso}$), 137.7 (q, superimposed **3a**$_{Phipso'}$ $_{+}$ **3b**$_{Phipso'}$), 134.3 (**3a**$_{Phpara}$), 134.1 (**3a**$_{Phpara'}$), 134.0 (**3b**$_{Phpara'}$), 131.3 (**3a**$_{Phortho'}$), 131.2 (**3b**$_{Phortho'}$), 129.6 (**3a**$_{Phortho}$), 129.3 (**3a**$_{Phmeta}$), 129.2 (**3b**$_{Phmeta'}$),129.1 (**3a**$_{Phmeta'}$), 128.5 (**3b**$_{Phmeta}$), 128.1 (**3b**$_{Phpara}$), 127.2 ($^{3}J_{CPt}$= 26.5, **3b**$_{Pymeta'}$), 127.1 ($^{3}J_{CPt}$= 25.7, **3b**$_{Pymeta}$), 127.0 ($^{3}J_{CPt}$= 27.4, **3a**$_{Pymeta}$), 125.3 (s, **3b**$_{Phortho}$), 103.0 (q, **3b**$_{C\text{-}OH}$).

^{195}Pt NMR (107 MHz, d_3-MeCN) δ: 689 (**3a**), 785 (**3b**).

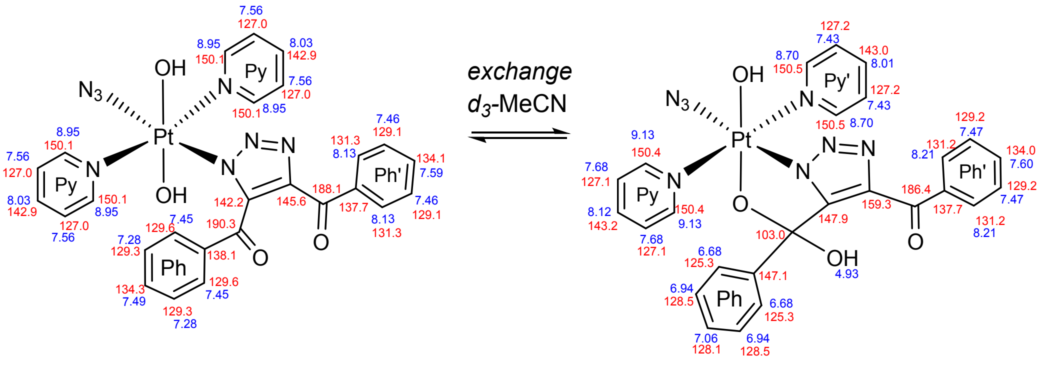

[3a] **[3b]**

Figure S3. NMR spectral assignments (^{1}H blue, ^{13}C red) for **3a** and **3b**.

ESI-MS, MS/MS and HRMS of complex 3 (3a/3b)

ESI-MS (MeCN) *m/z:* 1433.29 ([**3**$_2$+Na]$^+$, ([(Pt(N_3)($C_{16}H_{10}N_3O_2$)(OH)$_2$(py)$_2$)$_2$+Na]$^+$, $C_{52}H_{44}N_{16}NaO_8Pt_2$ calcd. 1433.27), 728.14 ([**3**+Na]$^+$ ([Pt(N_3)($C_{16}H_{10}N_3O_2$)(OH)$_2$(py)$_2$+Na]$^+$ $C_{26}H_{22}N_8NaO_4Pt$ calcd. 728.13), 688.15 ([**3** – OH]$^+$, [Pt(N_3)($C_{16}H_{10}N_3O_2$)(OH)(py)$_2$]$^+$, $C_{26}H_{21}N_8O_3Pt$, calcd. 688.14).

MS/MS (706.15) *m/z*: 688.140 ([**3** – OH]$^+$, [Pt(N_3)($C_{16}H_{10}N_3O_2$)(OH)(py)$_2$]$^+$, $C_{26}H_{21}N_8O_3Pt$, calcd. 688.134), 567.084 ([**3** - PhOH, N_3]$^+$, $C_{19}H_{26}N_5O_3Pt$, calcd. 567.168), 541.096 ([**3** - PhCO, N_3, OH]$^+$, $C_{19}H_{16}N_5O_2Pt$, calcd. 541.095), 370.055 ([Pt(OH)(py)$_2$]$^+$, $C_{10}H_{11}N_2OPt$ calcd. 370.055).

HRMS (MeCN) *r*

(MeCN) *m/z*: 70

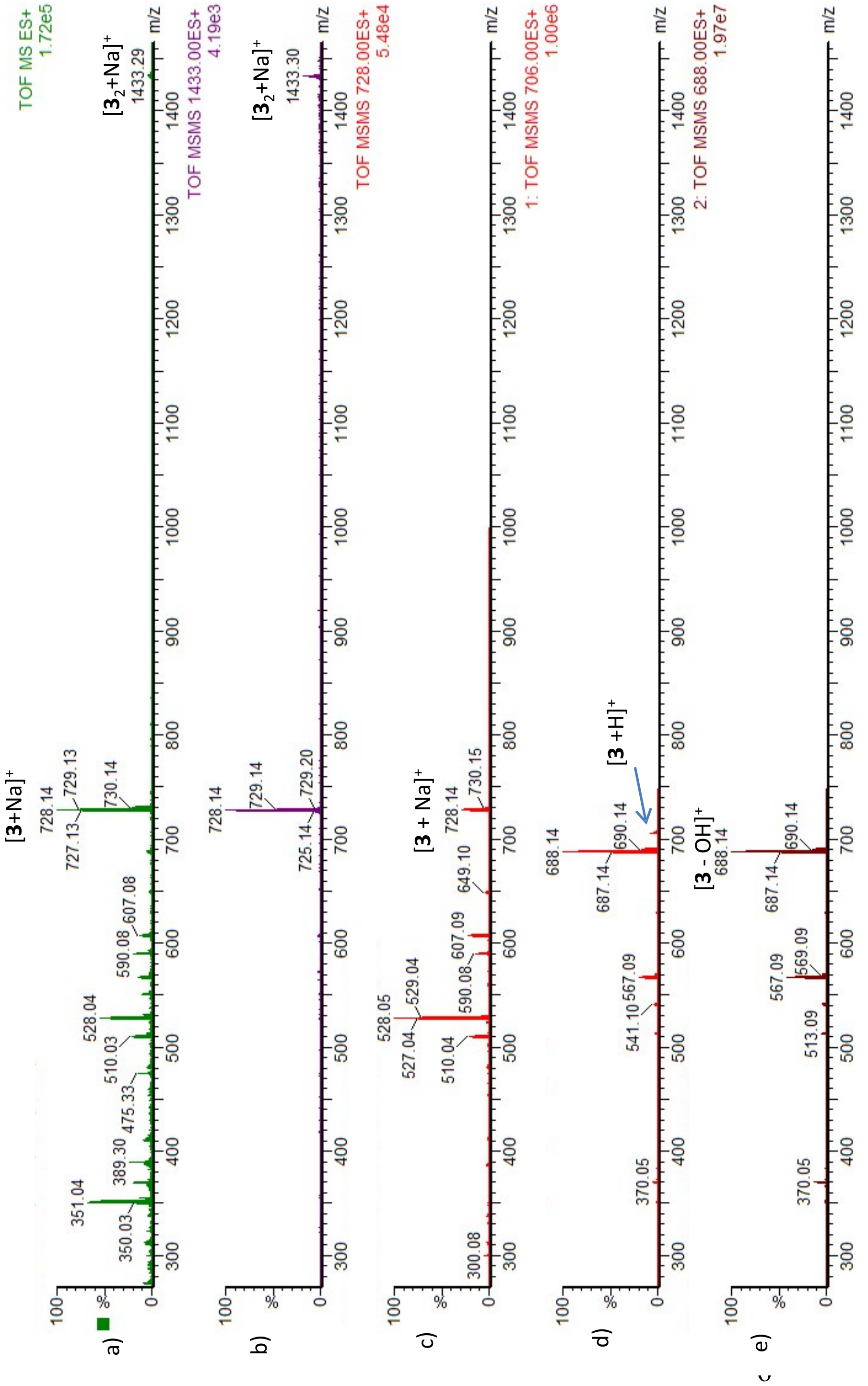

Figure S4. a) ESI-MS of complex **3** (MeCN). MS/MS fragmentation of species; b) [(**3**)$_2$ + Na]$^+$ (1433 *m/z*); c) [**3** + Na]$^+$ (728 *m/z*); d) [**3** + H]$^+$ (706 *m/z*) and e) [**3** - OH]$^+$ (688 *m/z*).

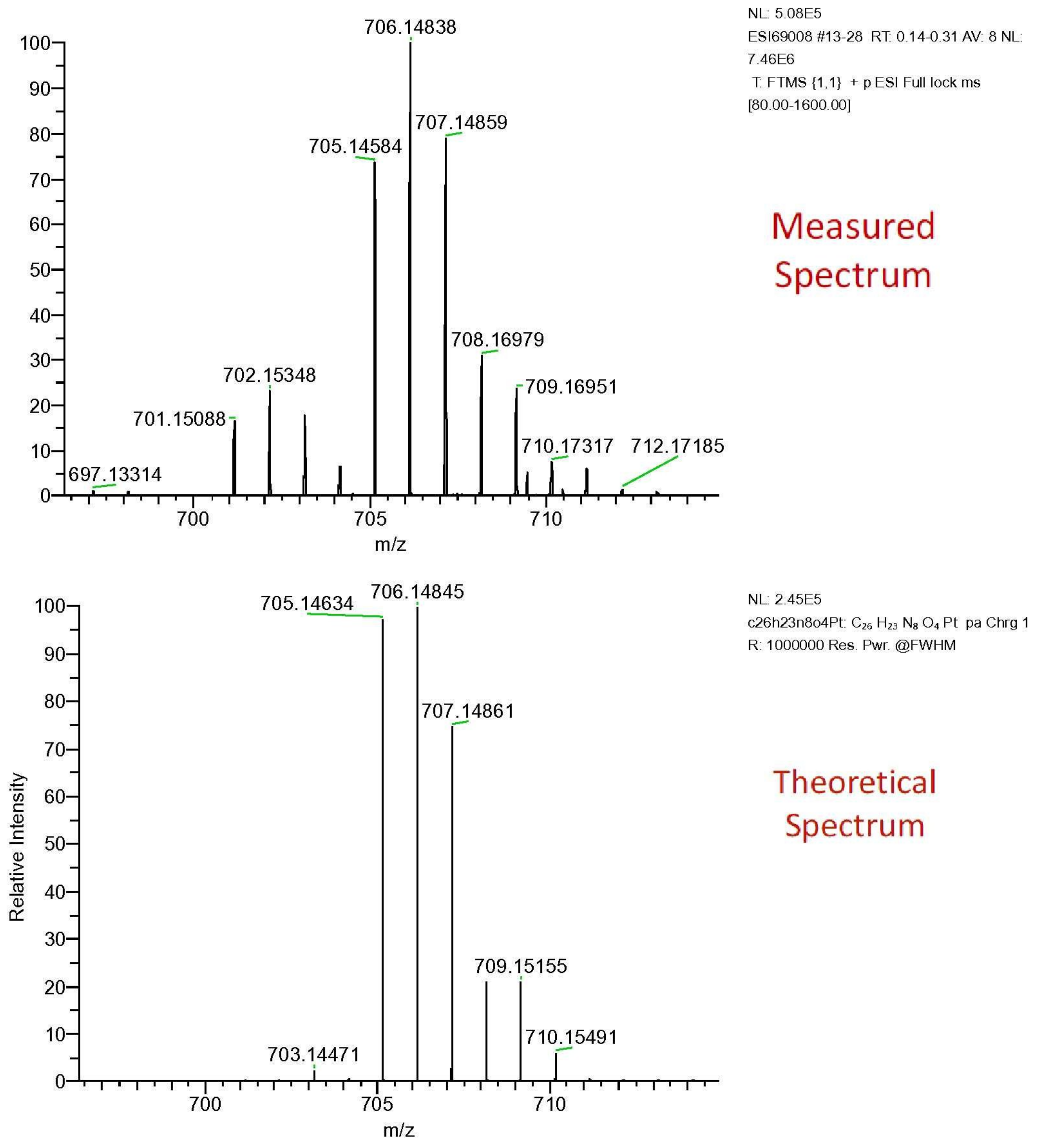

Figure S5. HRMS of complex **3** species [M+H]$^+$ (706.1484 *m/z*).

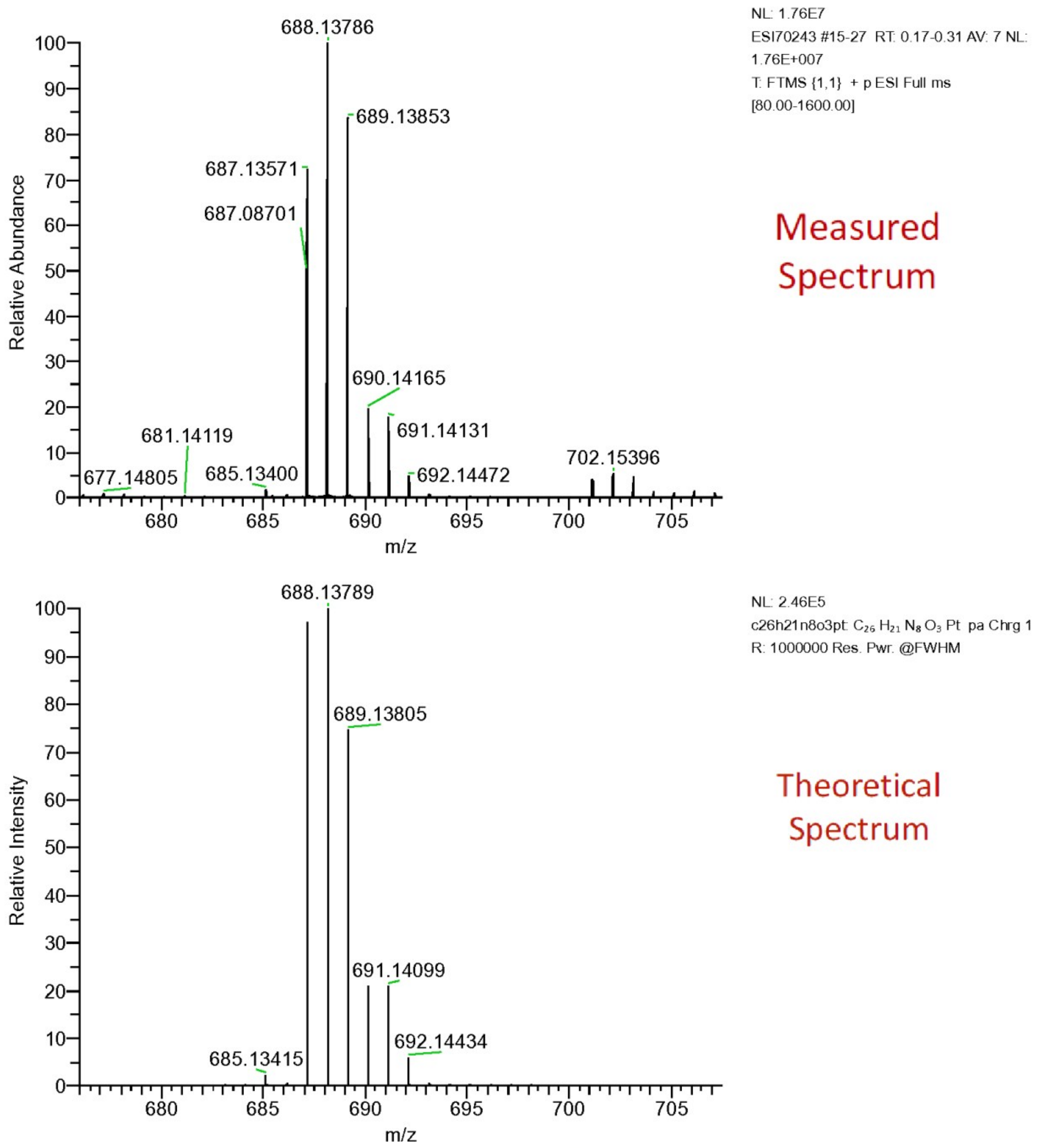

Figure S6. HRMS of complex **3** species [**3** – OH]$^+$ (688.1379 *m/z*).

UV-Vis spectra

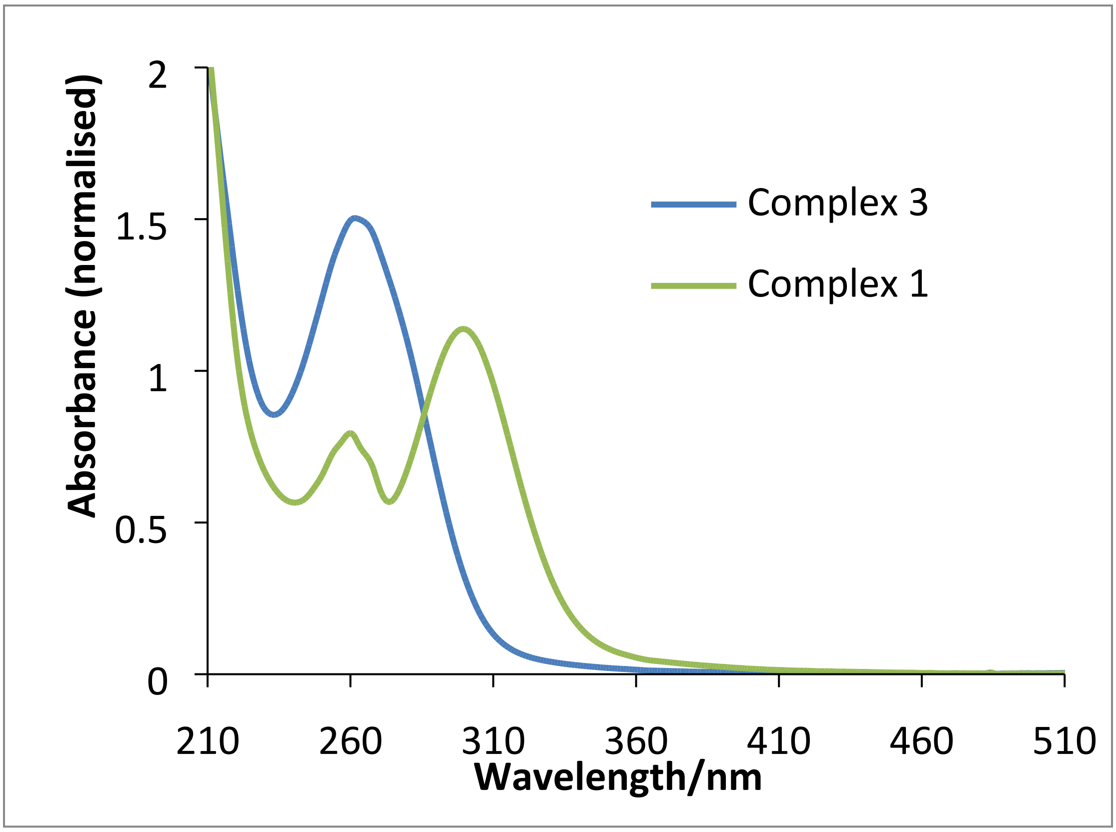

Figure S7. UV-Vis spectrum (H_2O/MeCN) of complex **1** and complex **3.**

IR spectra

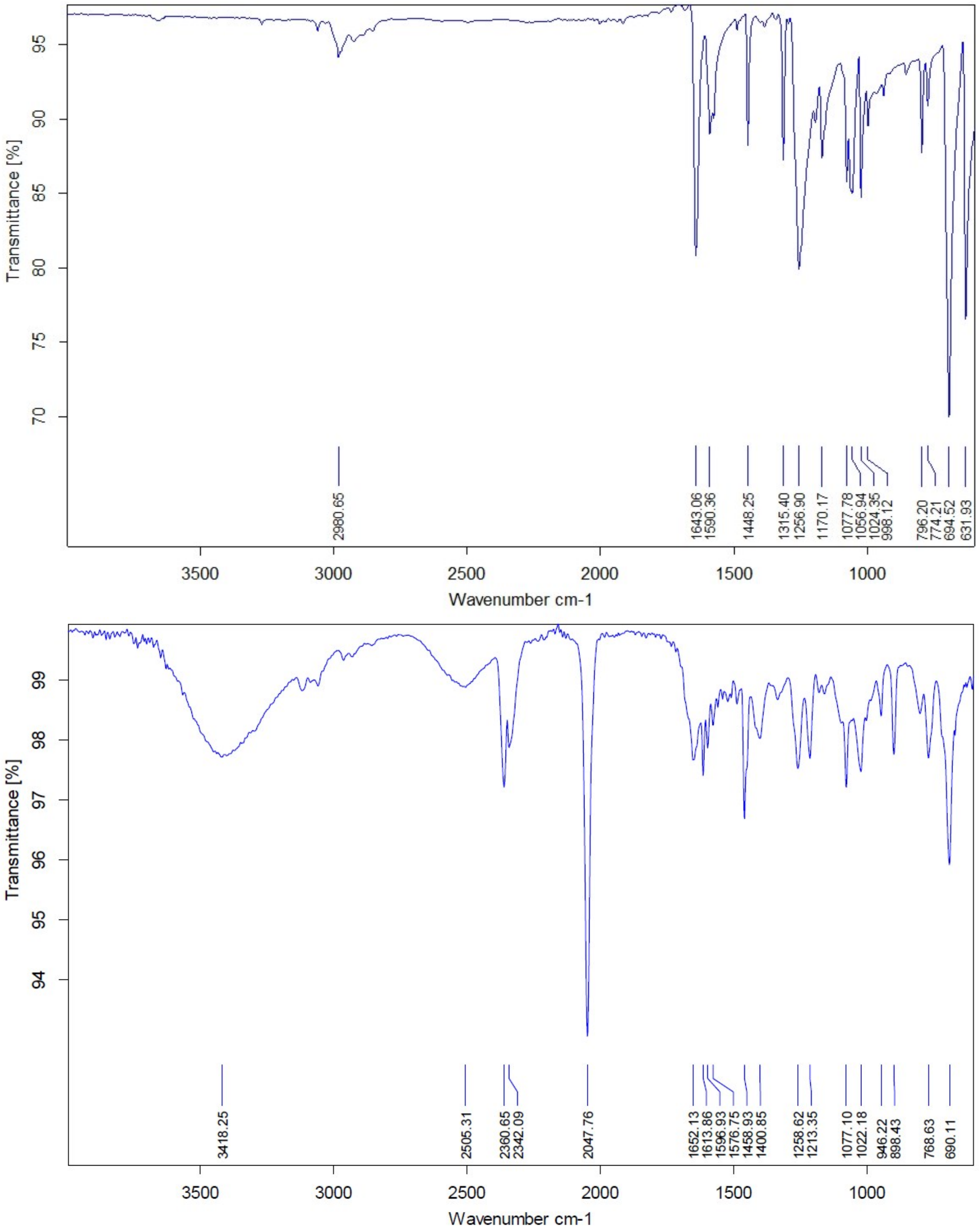

Figure S8. IR of a) starting material 1,4-diphenyl-2-butyne-1,4-dione (**2**) (solid) and b) complex **3** (d_4-MeOH).

NMR kinetics

The rate of exchange was measured using a selective saturation transfer experiment. A selective 180° pulse was used to invert the **3b** Ph_m resonance at 6.94 ppm. After a delay, a non-selective 90° was applied prior to acquisition of the ^{1}H NMR spectrum. By varying the delay the exchange between the **3b** Ph_m resonance at 6.94 ppm and the **3a** Ph_m resonance at 7.28ppm could be followed. This was repeated in 5K steps from 298K to 338K. The exchange rates at each temperature were calculated by fitting the build-up curves using the CIFIT program[1] and are summarised in the table below From the Eyring plot; $\Delta H^‡$ (kJ mol-1) = 25 (±1), $\Delta S^‡$ (J mol-1 K-1) = -173(±4) and $\Delta G^‡$ (298 K, kJ mol-1) = 76(±1) were derived (errors were calculated from the regression analysis of the Eyring plot).

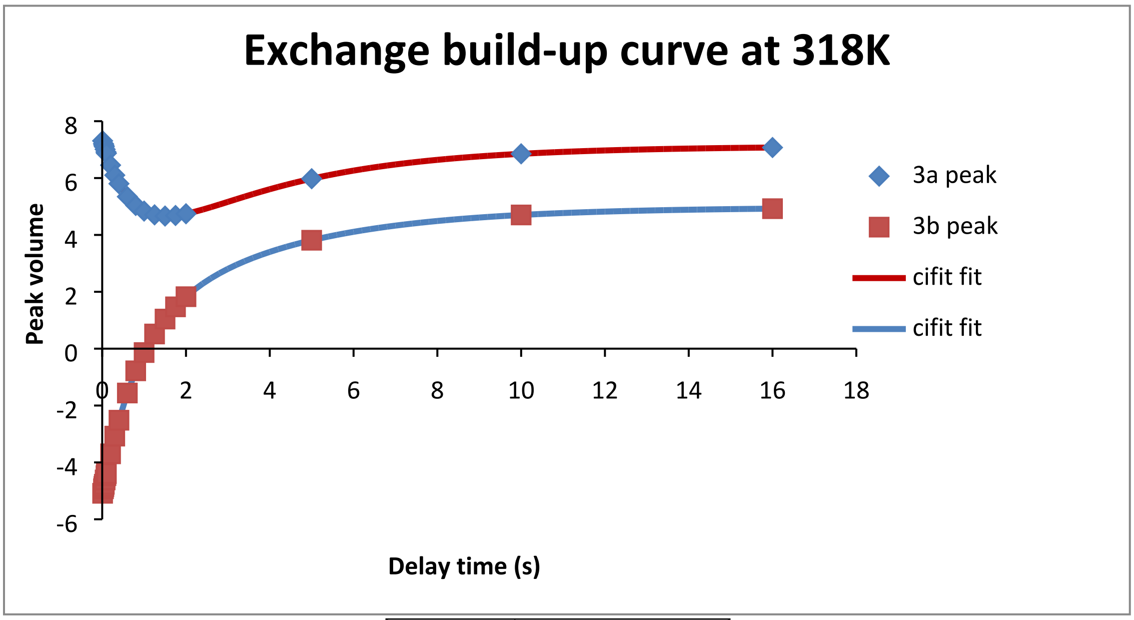

temp/K	Exchange rate
298	2.85E-01
303	0.3077
308	3.33E-01
313	4.23E-01
318	0.5041
323	6.29E-01
328	7.43E-01
333	8.07E-01
338	0.9836

Figure S9. Eyring plot and rate table for **3a** to **3b** interconversion kinetics.[3]

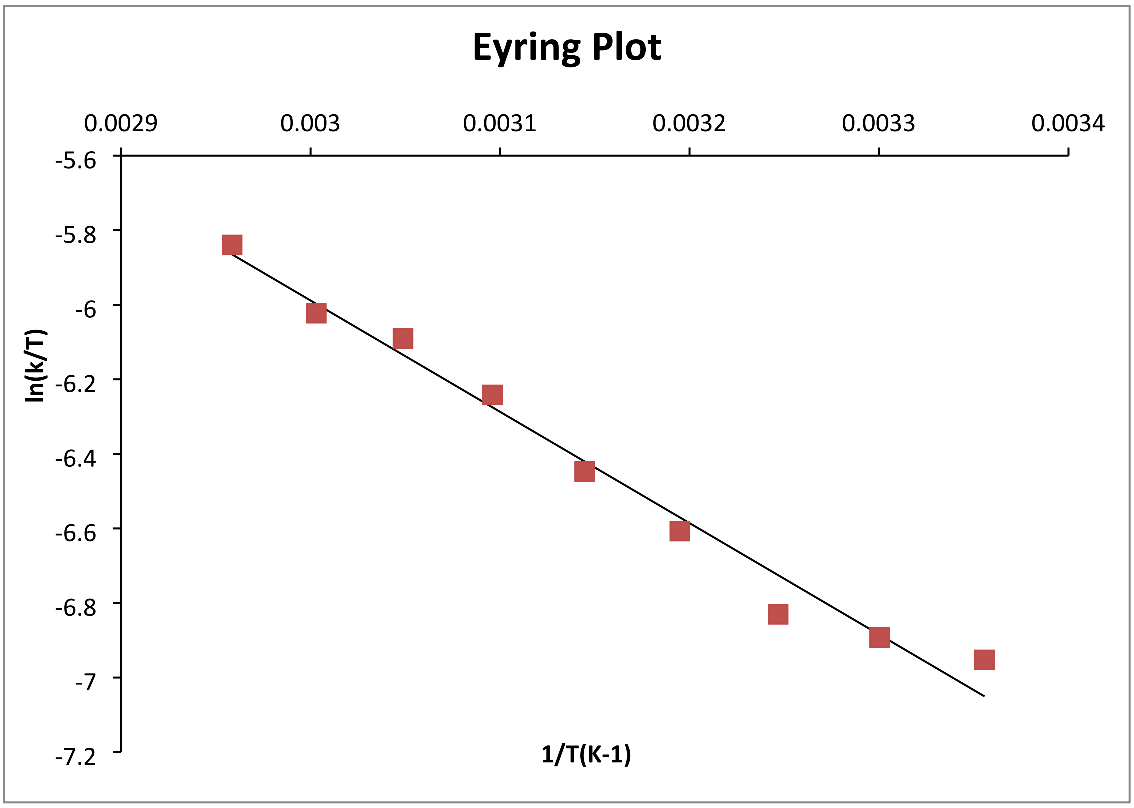

Figure S10. Build up Curve at 318 K, Exchange rate table for **3a** to **3b** interconversion kinetics and Eyring plot.

X-Ray crystallographic data and tables

X-ray crystallographic tables and figures were produced using Olex2.[4] Crystal Data for $C_{31}H_{32}N_8O_5Pt$ (3b.$C_5H_{10}O$) (M =791.73 g/mol): orthorhombic, space group Pbca (no. 61), a = 15.5699(6) Å, b = 13.6495(5) Å, c = 28.638(1) Å, V = 6086.3(4) Å^3, Z = 8, T = 100 K, μ(MoKα) = 4.666 mm^{-1}, D_{calc} = 1.728 g/cm^3, 20849 reflections measured (6.94° ≤ 2Θ ≤ 58.392°), 6965 unique (R_{int} = 0.0447, R_{sigma} = 0.0511) which were used in all calculations. The final R_1 was 0.0414 ($I > 2\sigma(I)$) and wR_2 was 0.0779 (all data).

**Table S1** Crystal data and structure refinement for 18KE08-1.	
Identification code	18KE08-1
Empirical formula	$C_{31}H_{32}N_8O_5Pt$
Formula weight	791.73
Temperature/K	100
Crystal system	orthorhombic
Space group	Pbca
a/Å	15.5699(6)
b/Å	13.6495(5)
c/Å	28.638(1)
α/°	90
β/°	90
γ/°	90
Volume/Å^3	6086.3(4)
Z	8
ρ_{calc}/g cm^{-3}	1.728
μ/mm^{-1}	4.666
F(000)	3136.0
Crystal size/mm^3	0.651 × 0.321 × 0.155
Radiation	MoKα (λ = 0.71073)
2Θ range for data collection/°	6.94 to 58.392
Index ranges	-19 ≤ h ≤ 19, -9 ≤ k ≤ 17, -31 ≤ l ≤ 39
Reflections collected	20849
Independent reflections	6965 [R_{int} = 0.0447, R_{sigma} = 0.0511]
Data/restraints/parameters	6965/0/408
Goodness-of-fit on F^2	1.116
Final R indexes [I>=2σ(I)]	R_1 = 0.0414, wR_2 = 0.0735
Final R indexes [all data]	R_1 = 0.0537, wR_2 = 0.0779
Largest diff. peak/hole / e Å^{-3}	1.69/-1.80

**Table S2**. Bond Lengths for 18KE08-1.						
**Atom**	**Atom**	**Length/Å**		**Atom**	**Atom**	**Length/Å**

Pt1	N1	1.998(4)		C26	C14	1.526(6)
Pt1	N3	2.034(4)		C26	C36	1.384(7)
Pt1	N15	2.035(4)		N18	C28	1.357(6)
Pt1	O5	2.017(3)		N18	C40	1.339(6)
Pt1	N18	2.031(4)		C23	C24	1.382(7)
Pt1	O6	1.982(3)		C23	C22	1.379(7)
N1	N13	1.360(5)		C14	C16	1.522(6)
N1	C16	1.340(6)		C14	O7	1.428(5)
O3	C5	1.224(6)		C21	C22	1.385(6)
C5	C11	1.474(6)		C28	C37	1.377(8)
C5	C20	1.491(7)		C32	C31	1.386(10)
C10	C20	1.401(8)		C40	C39	1.385(7)
C10	C29	1.391(7)		C33	C34	1.365(8)
C11	N14	1.375(6)		C39	C38	1.389(9)
C11	C16	1.379(7)		C34	C35	1.365(9)
N13	N14	1.314(5)		O43	C44	1.417(8)
C20	C32	1.396(8)		O43	C42	1.394(8)
N3	C25	1.348(6)		C37	C38	1.358(9)
N3	C21	1.345(6)		C36	C35	1.392(7)
N15	N16	1.218(6)		C29	C30	1.370(10)
O5	C14	1.397(5)		C30	C31	1.383(11)
N16	N17	1.142(6)		C44	C45	1.534(10)
C27	C26	1.382(7)		C41	C42	1.486(10)
C27	C33	1.393(7)		C41	C1	1.536(11)
C25	C24	1.382(7)		C45	C1	1.509(10)

Table S3. Bond Angles for 18KE08-1.

Atom	Atom	Atom	Angle/˚		Atom	Atom	Atom	Angle/˚
N1	Pt1	N3	89.28(15)		C27	C26	C14	123.1(4)
N1	Pt1	N15	172.21(17)		C27	C26	C36	118.6(5)
N1	Pt1	O5	80.95(15)		C36	C26	C14	117.8(4)
N1	Pt1	N18	91.41(16)		C28	N18	Pt1	120.6(4)
N3	Pt1	N15	89.63(16)		C40	N18	Pt1	119.3(3)
O5	Pt1	N3	86.62(15)		C40	N18	C28	120.1(4)
O5	Pt1	N15	91.28(15)		C22	C23	C24	119.2(5)
O5	Pt1	N18	88.73(14)		C25	C24	C23	119.3(5)
N18	Pt1	N3	175.13(16)		O5	C14	C26	107.3(4)
N18	Pt1	N15	89.04(17)		O5	C14	C16	109.9(4)
O6	Pt1	N1	100.95(15)		O5	C14	O7	106.1(4)
O6	Pt1	N3	94.32(16)		C16	C14	C26	113.8(4)
O6	Pt1	N15	86.83(16)		O7	C14	C26	111.8(4)
O6	Pt1	O5	177.88(14)		O7	C14	C16	107.6(4)
O6	Pt1	N18	90.28(16)		N3	C21	C22	120.7(5)

N13	N1	Pt1	134.0(3)		N1	C16	C11	105.3(4)
C16	N1	Pt1	115.5(3)		N1	C16	C14	116.8(4)
C16	N1	N13	110.5(4)		C11	C16	C14	137.8(4)
O3	C5	C11	118.5(5)		C23	C22	C21	119.5(5)
O3	C5	C20	120.6(4)		N18	C28	C37	120.3(6)
C11	C5	C20	120.9(5)		C31	C32	C20	120.1(7)
C29	C10	C20	120.3(6)		N18	C40	C39	120.9(5)
N14	C11	C5	124.4(4)		C34	C33	C27	120.3(5)
N14	C11	C16	108.1(4)		C40	C39	C38	119.1(6)
C16	C11	C5	127.4(4)		C35	C34	C33	119.6(5)
N14	N13	N1	107.8(4)		C42	O43	C44	108.4(6)
N13	N14	C11	108.3(4)		C38	C37	C28	120.3(6)
C10	C20	C5	124.0(5)		C26	C36	C35	120.0(5)
C32	C20	C5	116.8(5)		C30	C29	C10	119.6(7)
C32	C20	C10	119.0(5)		C34	C35	C36	120.9(6)
C25	N3	Pt1	116.8(3)		C29	C30	C31	121.1(7)
C21	N3	Pt1	122.6(3)		O43	C44	C45	112.2(6)
C21	N3	C25	120.3(4)		C37	C38	C39	119.2(5)
N16	N15	Pt1	114.2(3)		C42	C41	C1	108.1(7)
C14	O5	Pt1	116.6(3)		C30	C31	C32	119.8(7)
N17	N16	N15	174.8(6)		C1	C45	C44	109.7(7)
C26	C27	C33	120.6(5)		O43	C42	C41	114.5(6)
N3	C25	C24	120.9(5)		C45	C1	C41	110.6(6)

Photochemistry of 3

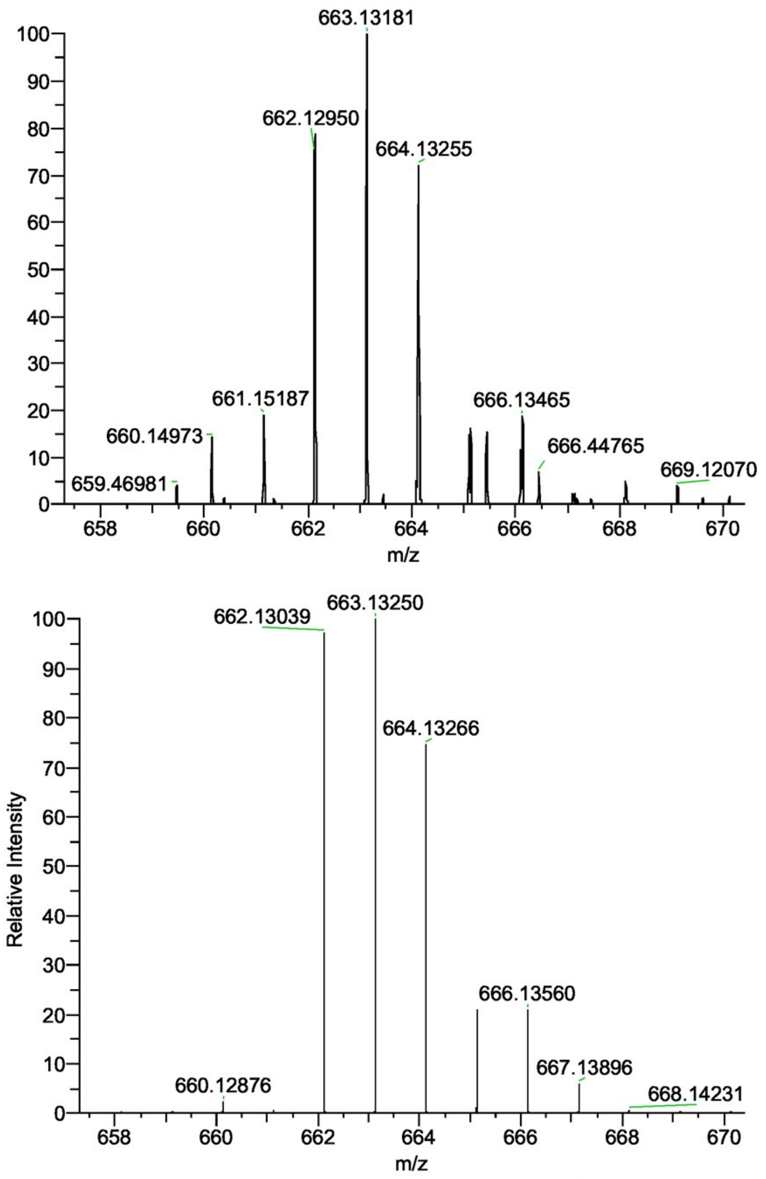

Figure S11. HRMS of photoproduct [**3**-N_3]$^+$: top (663.13181 *m/z* measured), bottom (663.13250 *m/z,* $C_{26}H_{22}N_5O_4Pt$ calculated), calc error -1.04 ppm.

EPR experimental data

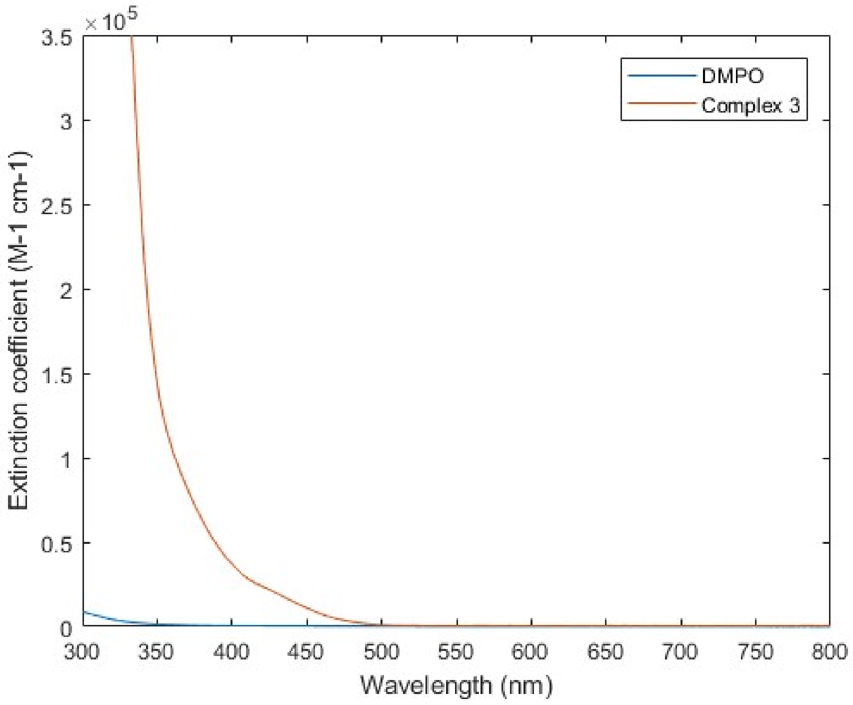

Figure S12. UV-Vis spectra (MeCN:H_2O 1:9) of concentrated sample of complex **3** (10 mM) and DMPO (10 mM) showing extent of absorption into the visible region of the spectrum, acquired a 1 mm quartz cuvette. Complex **3** shows some absorption in the short blue, while DMPO shows negligible absorption in the visible.

EPR sample preparation

DMPO was dissolved in MeCN at a concentration of 21 mM and was degassed in a Schlenk tube by performing 6 freeze-pump-thaw cycles, until no further gas bubbling was observed. The solution was then transferred to a N_2 glove-box and used to dissolve complex **3** (previously freeze-dried) to a concentration of 10.5 mM (with a molar ratio of DMPO:**3** of 2:1). This solution was pipetted into a 25 μL glass microcapillary (IntraMark, Blaubrand), and both ends were sealed with *cristaseal* sealing wax (Hawksley). Finally, the microcapillary was placed in an EPR quartz tube (4 mm external diameter) and the tube was sealed with a septum.

EPR experiments

The experimental setup consisted of a 1000 W Xe arc lamp coupled to the resonator (Bruker X-band Super High Sensitivity Probehead) of the EPR spectrometer (X-band EMXmicro, Bruker) through a liquid waveguide. The light was filtered before entering the waveguide, using a 410 nm long-pass filter followed by a 440 – 480 nm band pass filter. The measurement was carried out in continuous-wave mode, at X-band frequency (ca. 9.5 GHz) and room temperature. Fifty field scans of 8 mT width, with 30 s acquisition time per scan, were averaged to obtain each of the spectra plotted in figure S13. A receiver gain of 30 dB, modulation amplitude of 0.1 mT and microwave attenuation of 23.0 dB were used.

Quantification was done using the second integral of the spectra, by interpolation of a calibration curve of TEMPO solutions of known concentrations measured in the same conditions as the sample, in the dark. The steady state concentration of DMPO-$N_3^•$ was calculated from an averaged spectrum over the first 50 min of illumination, before the signal started to decay (Figure S14). For the final concentration of stable nitroxide, a spectrum averaged over 50 min recorded in the dark, after the DMPO-$N_3^•$ signal had fully decayed, was used.

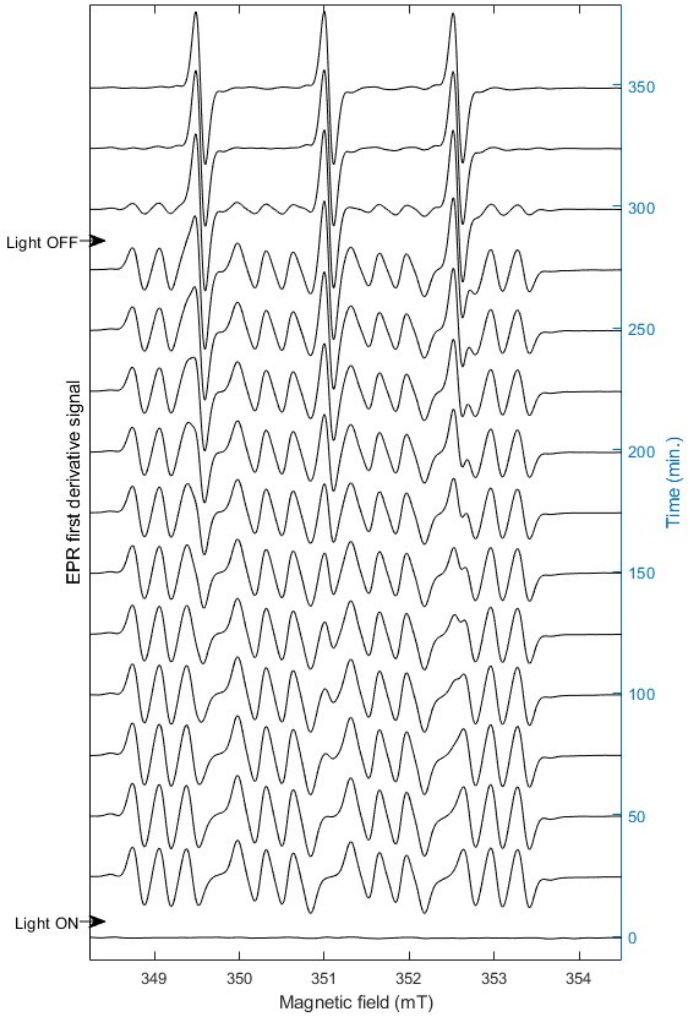

Figure S13. CW-EPR spectra showing the evolution of radicals from complex **3** under blue light illumination. Once the DMPO-$N_3^•$ had fully decayed after switching illumination off, only a very slow decay of the triplet signal was observed in the dark.

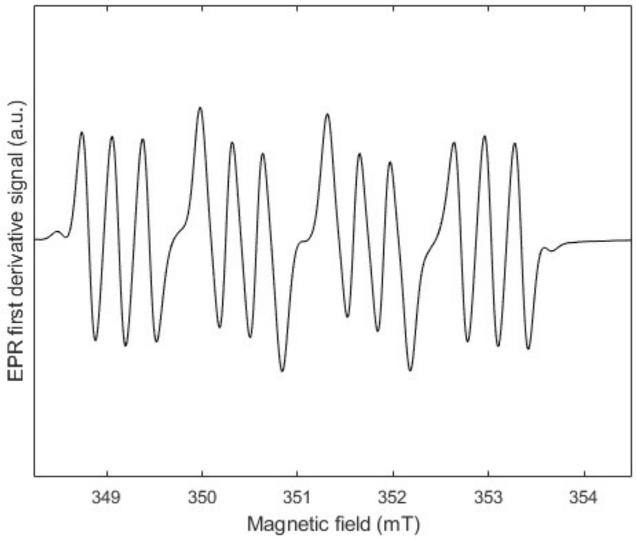

Figure S14. Average CW-EPR spectrum of complex **3** during the first 50 min of illumination, showing the characteristic multiplet structure of the DMPO-$N_3^{\bullet}$ adduct.

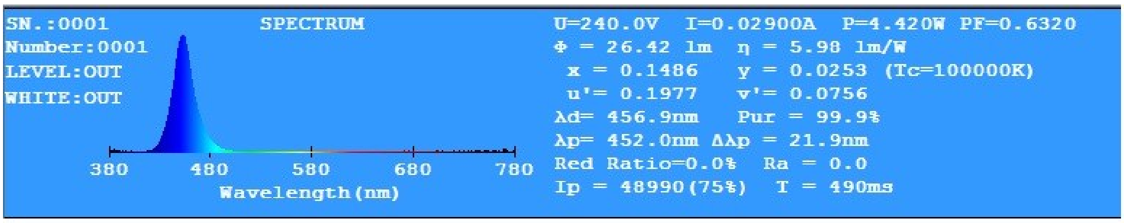

Figure S15. Spectral output of MiniSun (GU10 27 SMD) blue LED bulb, λ_{max} = 452 nm.

References

1 A. Bax and M. F. Summers, *J. Am. Chem. Soc.*, 1986, **108**, 2093–2094.
2 N. J. Farrer, J. A. Woods, L. Salassa, Y. Zhao, K. S. Robinson, G. Clarkson, F. S. Mackay and P. J. Sadler, *Angew. Chem. Int. Ed. Engl.*, 2010, **49**, 8905–8.
3 A. D. Bain and J. A. Cramer, *J. Magn. Reson. - Ser. A*, 1996, **118**, 21–27.
4 O. V. Dolomanov, L. J. Bourhis, R. J. Gildea, J. A. K. Howard and H. Puschmann, *J. Appl. Crystallogr.*, 2009, **42**, 339–341.
